# Otoendoscope combined with ablation electrodes for treatment of benign tracheal stenosis caused by granulation tissue hyperplasia after tracheotomy

**DOI:** 10.11604/pamj.2020.36.382.25125

**Published:** 2020-08-31

**Authors:** Laina Ndapewa Angula, Yongliang Teng, Le Sun, Xin Wang, Jing Shang, Ning Fang

**Affiliations:** 1 Department of Otolaryngology, First Hospital of Jilin University, Changchun, China

**Keywords:** Otoendoscopy, tracheal stenosis, tracheotomy, granulation tissue

## Abstract

Benign tracheal stenosis mainly appears due to tracheotomy, tuberculosis, trauma, benign tumor, or ventilation. With the increase in the number of tracheotomies and the prolongation of the life span of patients after incision, the long-term complications after tracheotomy gradually increase, among which intratracheal granulation hyperplasia is a more serious complication. The present case describes a 59-year-old male with granulation tissue hyperplasia induced by tracheotomy. He underwent tracheal resection to remove the granulation tissue and he remained well after the follow-up. Even though the endoscopic intervention and tracheal resection are readily accessible, they usually quite challenging. Here we summarize the present details on this condition.

## Introduction

Tracheal stenosis is considered as difficult due to complex field visualization and instrument narrowness. Coblation is preferred due to its fast and accurate ablation, less thermal damage *et al*.yet, more information is still required to demonstrate the ability of coblation in treating airway stenosis [1]. In our case, we report a unique technique using tracheotomy-coblation for managing tracheal stenosis that was caused by tracheotomy.

## Patient and observation

A 59-years-old male patient who suffered from cerebral hemorrhage nine years ago, underwent hematoma removal, decompressive craniectomy, and tracheostomy. He has a history of hypertension for 30 years, regularly taking amlodipine to control blood pressure; a history of diabetes for 7 to 8 years, taking acarbose for treatment. After the operation, he was given symptomatic and supportive treatment such as dehydration to lower intracranial pressure and anti-infection. He had left hemiplegia, speech difficulty, poor listening comprehension, most daily life dependent on the bed, and took soft food orally. Two months ago, the family members had difficulty suctioning and sucking sputum, and the trachea felt blocked of the patient. He was generally in good condition, with no fever, no dysphagia, no nausea or vomiting, normal urine and bowel movements, and no significant recent weight changes. Upon the visit to our hospital, a laryngoscope examination revealed a tracheal mass. Surgical treatment was recommended.

**Figure 1:**
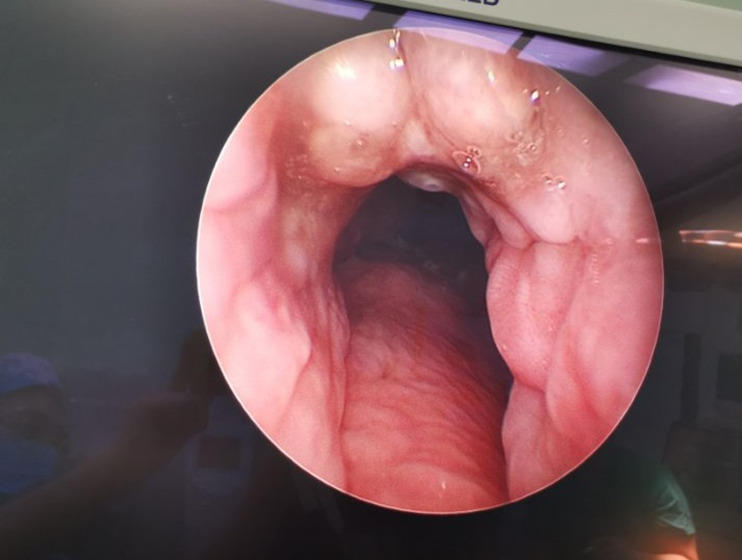
otoendoscopy showing a raised spherical granulation tissue observed on the anterior wall of the trachea with a cobblestone-like appearance

**Figure 2:**
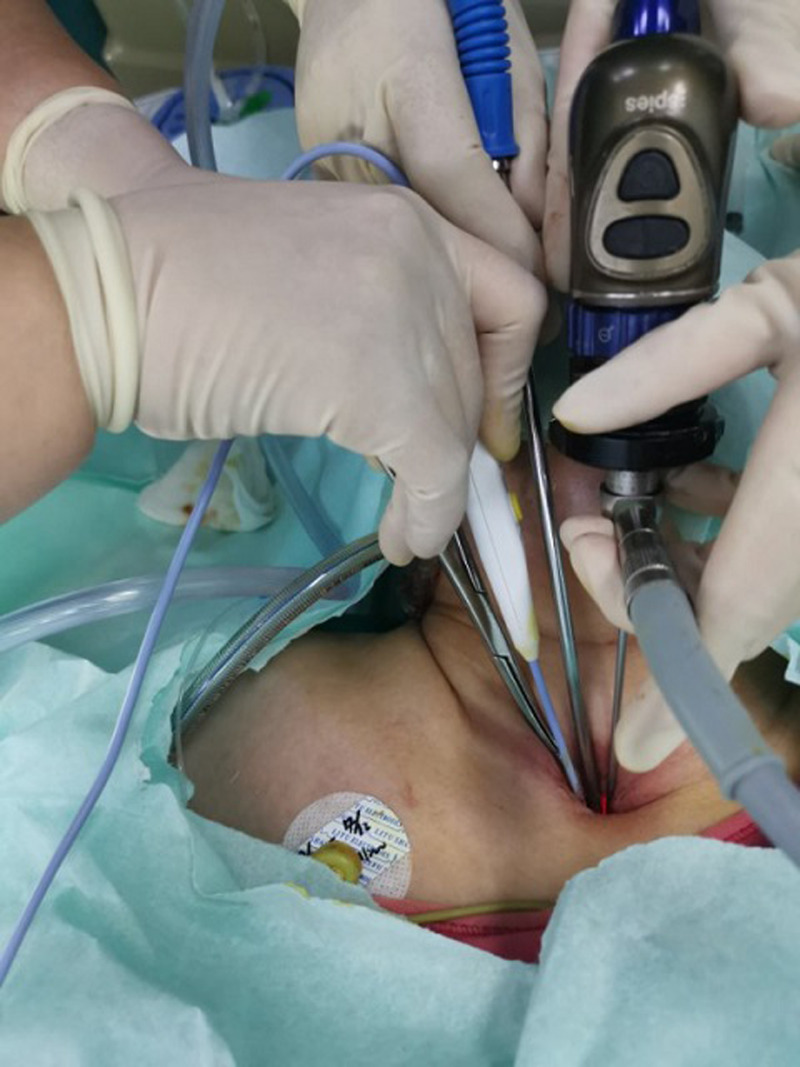
during the operation, the coblator in combination with unipolar and bipolar electrodes removes the tracheal granulation tissue

To perfect the preoperative examination, the patient lies in a supine position. After the general anesthesia takes effect, the head is tilted back, routinely disinfected, and a sterile sheet is laid. The blood oxygen saturation was maintained between 95% - 100%. The tracheal tube was removed with scar tissue at the tracheotomy opening. Tracheotomy opening was expanded with curved forceps, and otoendoscope is inserted. During the tracheal observation, a raised spherical granulation tissue was seen on the anterior wall of the trachea with a cobblestone-like appearance (Figure 1). Curettes are used to scraping off the tissue. The tissue had a tough texture with little bleeding. Then, electrocoagulation was done with alternate unipolar and bipolar electrodes (Figure 2). The granulation was ablated until the lumen was no longer obstructed, and no active bleeding was observed under the endoscopy. The tracheal resection was smooth, without side injury. The operation was completed. The granulation tissue was removed and sent for pathology. The postoperative pathology report revealed a fibrous fatty tissue covering squamous epithelium, fibrous tissue proliferation, more acute and chronic inflammatory cell infiltration, and small blood vessel proliferation. Upon follow up, the patient sucked sputum smoothly through the tracheal tube, and there was no sense of obstruction.

## Discussion

Benign tracheal stenosis caused by tracheotomy is a very challenging condition. Usual treatment includes endoscopic intervention and segmental tracheal resection. Anastomosis and traditional surgical resection have some drawbacks, for example, the extent of the resection. Apart from avoiding anastomotic stricture, the resection of the trachea also includes the abnormal stoma part and the part of the stenosis [2]. Tracheal stenosis can be managed through tracheal intervention [3], like coblation, tracheal stent, and laser. Many literature's have recommended that for the management of benign post-tracheotomy tracheal stenosis, tracheal resection is the more preferred modality with consideration to long-term results [4, 5]. We trust that the contraindications for tracheal resection are minimal. In previous literature, no patients were refused surgery for systemic rationale. In the group of patients who were refused surgery, this was chiefly because of glottic stenosis where it was believed that the outcome would not be beneficial.

One of the complications of tracheotomy is granulation tissue formation [6]. Granulation tissue may block the trachea at the level of the stoma and cause difficulty in sucking and suctioning sputum. As the granulation tissue matures, it develops into a fibrous and covered layer of squamous epithelium. With the maturation of fibrosis, stenosis appears as the lateral and anterior feature of the tracheal wall, narrowing at the stoma zone. Tracheal stenosis may develop at the site of the tracheal-tube cuff, where vascular injury to the submucosa of the trachea can arise when cuff pressure surpasses the coronary perfusion pressure of the capillaries of the wall of the tracheal. With delayed ischemia, epithelial ulcer, inflammation, and necrosis of cartilage may appear, resulting in the formation of granulation tissue. Shearing forces from the tube or the cuff may bruise the airway [7]. The granulation tissue in our patient may have appeared as a result of prolonged mucosal irritation and the injury due to repetitive monthly suction catheters. To avoid the formation of granulation tissue, methods have focused on preventing excess mechanical irritation. Many therapeutic methods have been detailed in patients with granulation tissue.

## Conclusion

Apart from the type of primary disease whether benign or neoplastic, tracheal resection has confirmed to be safe procedures and the good outcome rate is high. Even if complications develop, the morbidity rate is 45%. The tracheotomy-coblation seemed favorable with limited operation injury compared to traditional surgery and seemed safer and more appropriate under direct visualization. It needs only one operation which could facilitate the exhausting process of repeated ablation and lessen the patient´s concern.
